# Social Capital, Financial Literacy, and Rural Household Entrepreneurship: A Mediating Effect Analysis

**DOI:** 10.3389/fpsyg.2021.724605

**Published:** 2021-08-27

**Authors:** Jingmei Zhao, Tiancheng Li

**Affiliations:** ^1^School of Finance, Southwestern University of Finance and Economics, Chengdu, China; ^2^Research Institute of Economics and Management, Southwestern University of Finance and Economics, Chengdu, China

**Keywords:** social capital, household entrepreneurship, financial literacy, mediating effect, information communications technologies, rural China

## Abstract

In rural areas, entrepreneurship helps lift households out of poverty by alleviating unemployment and increasing income, and financial literacy plays an important role in promoting entrepreneurship. Social capital is a resource embedded in social relationships, the boundaries of which have been expanded by the development of information communications technologies (ICTs). This article aims to link social capital, financial literacy, and rural entrepreneurship through a partial mediating effect analysis. Using data from the 2015 China Household Finance Survey (CHFS), we analyze how social capital affects rural entrepreneurship and the role of local ICTs development in this effect while also accounting for reverse causality. We construct a social capital indicator, mainly referring to bridging social capital, and two financial literacy indicators to make the conclusions robust. The empirical results show that social capital promotes rural entrepreneurship by sharing financial literacy. Furthermore, the spread of ICTs enhances this mediating effect. Our study provides empirical evidence for encouraging entrepreneurship and promoting knowledge sharing and implies the importance of ICTs in promoting entrepreneurship in rural areas.

## Introduction

Rural areas are facing the challenges of slow development and population decline. Rural development is a vicious cycle in which a lack of a critical mass of services and infrastructure leads to a lower rate of business creation, and fewer job opportunities cause out-migration and aging (Paniagua, 2013; Pato and Teixeira, 2016). Consequently, more rural families are migrating to cities considering better public services and employment opportunities, which brings congestion, corruption and poverty to urban areas (Musemwa, 2010; Boohene and Agyapong, 2017). Rural entrepreneurship or self-employment (hereafter, entrepreneurship) has become part of the agenda of some governments and institutions (e.g., OECD, FAO, and UN) as an important factor in promoting rural development. Entrepreneurship refers to the conversion of existing opportunities to create future goods and services (Rindova et al., 2009). Rural entrepreneurship contributes to creating jobs and increasing income by enabling households to participate in income-generating activities (Kijima et al., 2006; Naminse and Zhuang, 2018). In this sense, ways of encouraging rural entrepreneurship have become an extremely important issue. The literature has found that households’ financial knowledge is positively correlated with entrepreneurial activity. Financial knowledge is a measure of the ability to understand and use economic and financial information. Delavande et al. (2008); Jappelli and Padula (2013), and Lusardi et al. (2017) argue that financial knowledge is a type of human capital and that it is one of the indicators of an individual’s ability. A lack of financial knowledge makes it difficult to identify and understand information about returns, risks, and financial products and to process such information. This difficulty explains household financial decision-making behaviors such as asset allocation, retirement planning (Lusardi et al., 2017, 2018), and limited participation in the stock market (Van Rooij et al., 2011). The entrepreneurial behavior of households includes the stages of identifying entrepreneurial opportunities, integrating entrepreneurial resources and operating a business. Each of these stages requires a great deal of time and effort to search for information, analyze the collected information effectively and use it wisely to make entrepreneurial decisions. These steps inevitably involve financial issues. Therefore, entrepreneurs need to be financially literate, and financial literacy has a positive effect on business development (Garg and Singh, 2018; Abad-Segura and González-Zamar, 2019; Xu et al., 2020; Burchi et al., 2021). Improving rural households’ financial literacy is an effective way to promote entrepreneurship.

Studies have shown a positive link between social capital and entrepreneurship, with social capital helping in developing entrepreneurial motivation, identifying business opportunities, and accessing entrepreneurial resources (Fuentes-Fuentes et al., 2015; Liu et al., 2019; Trigkas et al., 2020). Social capital is considered to be a resource embedded in social relationships (Lin, 2002). Land limits the relationships between households, especially in rural areas. Relationship-based social capital has important effects on rural household production, such as accelerated responses to climate change (Carter and Maluccio, 2003), the diffuse application of new technologies (Munasib and Jordan, 2011) and land management (Nath et al., 2010). Social capital also helps individuals find a job (Ge and Wu, 2020), increase their income (Yuan, 2016) and enhance life satisfaction (Lim and Putnam, 2010). In rural areas where physical and human capital are relatively scarce, social capital becomes an important resource for rural households. Additionally, social capital is an important channel through which rural families share knowledge (Kadushin, 2012; Martini et al., 2017). However, few studies have attempted to link social capital, financial literacy, and rural entrepreneurship. Furthermore, the usage of information communications technologies (ICTs) changes the way people communicate and may affect the role of social capital. ICTs shorten the distance between people, and this compressed world facilitates the flow of people, capital, and culture (Burnett and Marshall, 2003). In rural areas in particular, remote locations and inconvenient transportation hinder people’s interactions. Using ICTs, individuals can communicate remotely and online, which greatly contributes to the creation of new social networks. However, ICT-based connections lack social trust (Townsend et al., 2016). The impact of ICTs on social capital has not been well understood. This article attempts to explore how rural households use social capital with the support of ICTs.

Social capital is an important resource for rural households, and how rural households use social capital to start their own businesses has been a hot topic in current research (Evansluong and Ramírez-Pasillas, 2019; Liu et al., 2019; Trigkas et al., 2020). Different from them, we focus on the impact of social capital on rural entrepreneurship and introduce financial literacy into the mechanism study. We construct a social capital indicator that is mainly based on bridging social capital, which provides more advantages in terms of access to information. We also construct a financial literacy indicator based on households’ answers to financial questions. We then evaluate how social capital affects entrepreneurship and the partial mediating effect of financial literacy on this relationship. We find that social capital promotes rural entrepreneurship by sharing financial literacy. Furthermore, by dividing the level of ICTs adoption across regions, we also find that ICTs enhance this mediating effect.

Endogeneity issues may occur and lead to biased results when entrepreneurship inversely affects household social capital. We thus adopt the average social capital of households in the same community as an instrumental variable because entrepreneurship does not affect the social capital of other households in the community. Our result is robust after considering reverse causality.

This paper contributes to the existing literature in two ways. First, our study enriches the literature about the impact of social capital on rural entrepreneurship. Previous studies in this field have mainly focused on addressing households’ liquidity constraints (Cai et al., 2018; Sun et al., 2018) and the identification of entrepreneurial opportunities (Shu et al., 2018; Evansluong and Ramírez-Pasillas, 2019), while this paper finds that social capital promotes entrepreneurship through sharing financial literacy from the perspective of knowledge sharing. This explanation has important implications for understanding rural knowledge-sharing mechanisms and achieving sustainable development in rural areas. Second, we extend the impact of ICTs on social capital to rural entrepreneurship. Previous literature has found that ICTs enrich social capital networks, which makes it easier for people to access information in social networks (Townsend et al., 2016; He and Li, 2019). In this paper, we apply this positive effect to encourage rural entrepreneurship. This implies the important role of ICTs in promoting entrepreneurship in rural areas.

## Theoretical Background and Hypothesis Development

### Social Capital and Rural Entrepreneurship

Social capital is a sociological concept that refers to “social networks, trust and norms that can improve economic efficiency through coordinated action” (Putnam and Leonardi, 1994). In a traditionally relational society (Bian, 1997), social capital has been pivotal in determining the socioeconomic status of households. Social capital is considered to be one of the most important factors influencing household economic activity, as it can share risks, smooth consumption, promote employment, and reduce income inequality (Grootaert, 1999; Munshi and Rosenzweig, 2006, 2009; Kinnan and Townsend, 2012). In rural areas, widespread social capital is an important resource for entrepreneurs. For private enterprises, the social capital formed through political relations helps enterprises obtain policy resources, such as a larger proportion of government subsidies or entry into a regulated industry. Despite comprehensive economic reforms and the establishment of a modern market economy, doing business still requires business connections (Putnam and Leonardi, 1994). The positive role of social networks in household entrepreneurship has been discussed by most of the literature.

The literature has argued that social capital promotes rural entrepreneurship through financial availability and information accessibility. When private enterprises are in the earliest stages of existence, it is difficult for them to obtain support from formal institutions, while informal culture and tradition are particularly important (Xin and Pearce, 1994; Casson and Giusta, 2007) because rural households lack collateral, which reduces the possibility of rural household loans (Ahlstrom and Bruton, 2010; Schmalz et al., 2017; Sadeghloo et al., 2018). Rural households rely more on private financing (Jia et al., 2013; Hu and Zhang, 2014). Social networks can effectively alleviate information asymmetry in the financial market (Ghatak, 1999; Karlan, 2007). Because the members of social networks are often linked based on blood, geography and kinship ties, and the cost of supervision is low. Borrowers with higher risk can be easily identified and excluded from the private lending market (Karlan, 2007; Sun et al., 2018). Social networks can also act as an implicit guarantee mechanism so that defaulters suffer reputational damage, thus greatly reducing the likelihood of default in the private lending market (Karlan and Morduch, 2010).

In addition to financing channels, social capital can also influence entrepreneurship through information channels. An important part of the process of identifying entrepreneurial opportunities is the process of acquiring and screening entrepreneurial information. Based on this information, potential entrepreneurs need to measure benefits and costs in advance. Hence, the ability to access market information is a necessary skill for entrepreneurs (Simon et al., 2000). Social capital helps entrepreneurs broaden their information sources and improve the quality, accuracy, and timeliness of the information obtained (Coleman, 1988; Lin, 2002; Uzzi and Gillespie, 2002). Daily communication maintains the social relationships of independent individuals in social networks and provides entrepreneurial information resources (Evansluong and Ramírez-Pasillas, 2019). When potential entrepreneurs deliberately seek entrepreneurial opportunities, social capital can also help reduce search costs because it entails corresponding reciprocity and obligation (Granovetter, 2005).

### Social Capital and Financial Literacy

From the perspective of network strength, social networks play a role in sharing knowledge. Strong ties can bring more high-quality information. Long-term and stable relationships can greatly reduce the cost of information acquisition, but individuals obtain convergent information from strong ties. With the establishment and development of enterprises, weak ties help entrepreneurs access specific knowledge and necessary information that is not available in closed social networks. And weak ties provide opportunities to communicate with people with relevant knowledge, and mutual trust promotes the correct interpretation of others’ knowledge (Akhavan and Hosseini, 2016; Ganguly et al., 2019). Especially when enterprises enter the early stage of development, weak ties act as a bridge between different groups and promote the flow of information. Personal social networks can provide access to knowledge that is not currently available (Arenius and De Clercq, 2005).

As special knowledge, financial literacy has a positive impact on entrepreneurship (Rugimbana and Oseifuah, 2010; Wise, 2013; Abubakar, 2015; Calcagno et al., 2020). Social capital can affect household financial literacy in two ways. First, the “peer effect” exists in social networks. Intervention and reinforcement from friends can lead to the development of appropriate behavioral habits (Gioia, 2017). Individuals usually imitate and learn from the behavior of other individuals in the social network, and the larger the social network in which the family is located, the more financial information it receives passively and actively, and the greater the probability of learning to acquire financial knowledge. For example, social networks influence investors’ motivation to learn financial knowledge and their willingness to invest (Xia et al., 2014). Second, the “Matthew effect” exists in social networks. When financial events occur, households will actively learn from the logic and analytical skills of financially literate individuals in social networks to form their own opinions (Gao et al., 2019). These findings lead to the following hypotheses:

**Hypothesis 1:** Social capital promotes entrepreneurship by sharing financial literacy in rural areas.

### Effect of ICTs on Social Capital

Information communications technologies provide new ways to develop social capital by removing the time and space barriers to communication (Galloway and Mochrie, 2005; Hansson et al., 2007; Wallace, 2012). Individuals are limited by geographic location and transportation in rural areas, and social networks face the barrier of distance, which limits individuals’ ability to access information through social capital. Individuals use digital technologies to bridge time and distance through phone, e-mail and websites. Although some studies have argued that ICTs are not as effective as face-to-face communication, individuals are still able to establish initial relationships through ICTs and then take the next step to off-line communication (Kujath, 2011). Some studies found low levels of ICTs adoption in rural areas, considering the cost of time needed to change habits and learn skills (Warren, 2007; Townsend et al., 2013). As ICTs become more widespread, individuals will enjoy the benefits. ICTs provide social media to encourage entrepreneurs to interact and thereby broaden their social networks (He and Li, 2019; Yin et al., 2019). Thus, ICTs enrich social networks and enhance the ability of social capital to provide information. These findings lead to the following hypothesis:

**Hypothesis 2:** The mediating effect of social capital promoting rural entrepreneurship through financial literacy is stronger in regions with higher levels of ICTs adoption.

## Materials and Methods

### Sample and Data Collection

The data come from the China Household Finance Survey (CHFS) conducted in 2015. This survey was developed by Southwestern University of Finance and Economics to create a database to investigate the financial behavior of Chinese households. The survey targeted 37,263 households in 29 provinces excluding Tibet, Xinjiang, Hong Kong, Macao and Taiwan. The data were collected from 29 provinces, 353 cities/counties, and 1,373 villages in all areas of China in 2015. The sampling was performed according to the principle of uniform sample selection in three stages and using the probability proportional to size (PPS) sampling method. The primary units of interest were 2,585 cities/counties in China (excluding Tibet, Xinjiang, Inner Mongolia and Hong Kong and Macao). The first stage was to select 353 cities/counties from 2,585 cities/counties in China following the principle of uniform geographical distribution and uniform economic development. The second stage was to randomly select the neighborhood committee/village committee from the city/county directly. Finally, households that were interviewed were randomly selected from the list of residents of a given neighborhood committee/village committee. The head of the household, as the respondent, was asked to answer a questionnaire including items related to demographic characteristics, assets and liabilities, insurance and social security, household expenditures and income, and views on family, marriage, and community governance. The head of the household is the owner of the property of the house and is the family member who knows the most about the household’s financial situation. The sample was divided into urban and rural areas according to administrative regions. We only use observations in rural areas, so the final sample consisted of 11,654 households. In addition, the ICTs adoption data come from the 2014 industrialization and informatization development level assessment report released by China’s Ministry of Industry and Information Technology.

### Variables and Measures

#### Social Capital

The main variable is the social capital held by a family. Previous studies give us guidelines on selecting variables to construct the social capital index. Social capital can be divided into bonding capital and bridging capital (Woolcock, 1998). Bonding capital refers to resources contained in small groups between blood relations, neighbors, and close friends. It emphasizes obligatory relationships and may lead to the exclusion of wider relationships. Bridging social capital refers to resources contained in wider groups and is not limited by geographic location. The difference between bonding capital and bridging capital is similar to that between strong ties and weak ties. Bonding social capital and bridging social capital may not be mutually exclusive but instead simply two aspects of social capital (Anderson and Jack, 2002; Phillipson et al., 2006). The social capital indicator we constructed refers to bridging capital, which offers advantages over bonding capital in accessing information (Coleman, 1988). Households obtain bridging social capital by creating and maintaining new relationships. Developing relationships requires a certain amount of expenditure, which can be seen as a cost or an investment in relationships. A common method of developing and maintaining relationships is to give gifts or to host recreational activities (Hwang, 1987). Different relationship bases correspond to different principles of interaction. An unconditional protective relationship between individuals with close kinship ties is provided without reciprocity. However, individuals in long-distance relationships usually consider the costs and expected rewards when offering help (Farh et al., 1998). Thus, we considered five household expenditure variables: expenditures on communication, transportation, dining, entertainment and gifts. The definitions of the selected variables are shown in Table 1. We choose the sum of these expenditures as a proxy for social capital.

**TABLE 1:** Composition of the social capital.

**Variable**	**Definition**
Communication expenditure	The average monthly expenditure on mobile phones, telephones and other communications used by the household members last year
Transportation expenditure	The average monthly local transportation expenditure for the family last year
Dining expenditure	The average monthly expenditure on dining out last year
Entertainment expenditure	The average monthly expenditure on TV, the Internet, and other entertainment-related activities last year
Gift expenditure	Transfers to non-family members last year, including spending on holidays and at weddings and funerals
	

#### Financial Literacy

The 2015 CHFS asked respondents three questions about interest rates, inflation, and risk awareness (see Table 2). We construct two measures of financial literacy. First, a financial literacy score is generated based on the number of questions that the respondents answered correctly. Second, we consider wrong answers and indirect answers (such as “I do not know” or “I cannot figure it out”) to represent different levels of financial literacy. Therefore, we construct two binary variables for each question. The first binary variable indicates whether the question was answered directly. The second binary variable indicates whether the question was answered correctly. As a result, we generate six binary variables. Following Van Rooij et al. (2011), we use principal component analysis to construct the financial literacy index.

**TABLE 2:** Questions in the 2015 CHFS related to financial literacy.

**Given a 4% interest rate, how much would you have in total after one year if you have 100 yuan deposited?**
1. Under 104
2. 104
3. Over 104
4. I cannot figure it out
With an interest rate of 5% and an inflation rate of 3%, the stuff you buy with the money you have saved in the bank for 1 year is:
1. More than last year
2. The same as last year
3. Less than last year
4. I cannot figure it out
Which one do you think is riskier, stock or a fund?
1. Stock
2. A fund
3. I haven’t heard about stock
4. I haven’t heard about funds
5. I haven’t heard about either stock or funds

#### Entrepreneurship

The dependent variable is household entrepreneurship. It is a dummy variable equal to 1 if any family member chose to be an entrepreneur. We defined this variable using the head of the household responses to the question “Is your family engaged in production and operation of industry and commerce, including individual business, leasing, transportation, online stores, and enterprises?” The dependent variable is equal to 1 if the respondent answered “Yes” and 0 otherwise if the respondent answered “No.” In this paper, we focus on non-farm entrepreneurship.

### Method of Analysis

**FIGURE 1:**
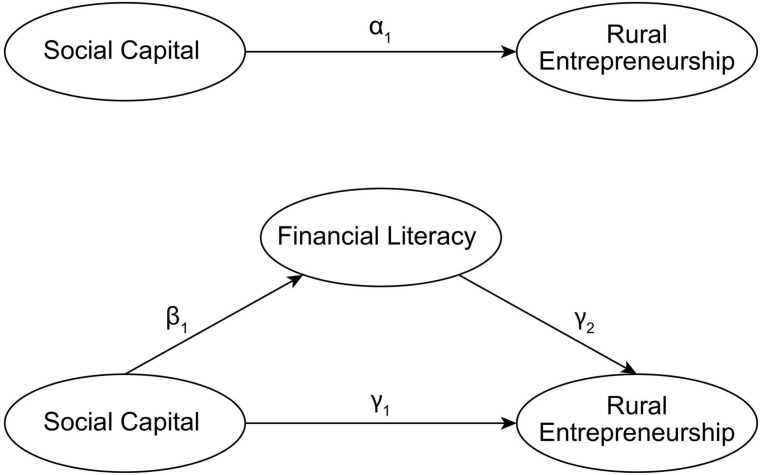
Relationships between Social Capital, Financial Literacy and Rural Entrepreneurship.

To identify the mediating effect of financial literacy on the influence of social capital on household entrepreneurship, we follow the approach from Taylor et al. (2008). First, to identify the relationship between social capital and entrepreneurship, the model is specified as in Equation (1). Second, we identify the relationship between social capital and financial literacy, and the model is specified as in Equation (2). Third, we add financial literacy as an explanatory variable to Equation (1), and the model is specified as in Equation (3). We use a probit model for estimation when the dependent variable is entrepreneurship, an ordered probit model when the dependent variable is the financial literacy score and an ordinary least squares (OLS) model when the dependent variable is the financial literacy index. To alleviate heteroscedasticity, we use White robust standard deviation estimation. If social capital contributes to rural entrepreneurship, then α_1_ = 0; if Hypothesis 1 holds, then β_1_ = 0 and γ_2_ = 0. If γ_1_ = 0, then we verify a partial mediating effect. The relationships of the main variables verified by our model are shown in Figure 1. The model is specified as follows:

P(Etrepreneurship=1)

(1) $$=\Phi \,\left(\alpha_{0}+\alpha_{1}\,S\,o\,c\,i\,a\,l\,C\,p\,i\,t\,a\,l+\alpha_{2}\,C\,o\,n\,t\,r\,o\,l+F\,E+\mu_{1}\right)$$

F⁢i⁢n⁢a⁢n⁢c⁢i⁢a⁢l⁢L⁢i⁢t⁢e⁢r⁢a⁢c⁢y

(2) $$=\beta_{0}+\beta_{1}\,S\,o\,c\,i\,a\,l\,C\,a\,p\,i\,t\,a\,l+\beta_{2}\,C\,o\,n\,t\,r\,o\,l+F\,E+\mu_{2}$$

P(Entrepreneurship=1)=

$$\Phi (\gamma_{0}+\gamma_{1}SocialCapital+\gamma_{2}FinancialLiteracy$$

(3) $$+\gamma_{3}Control+FE+\mu_{3})$$

Where, *Control* represents a vector of characteristics of the head of household and characteristics of the household. The characteristics of the head of household include gender, age, educational level, marital status and risk appetite. The characteristics of the household include family size, the number of seniors and kids in the family, a servant dummy, household net assets, and household net income. In addition, we control for provincial fixed effects to capture factors such as local policy and social conventions. The definitions of the variables are shown in Table 3. It is necessary to clarify that we focus on household entrepreneurship rather than individual entrepreneurship because entrepreneurial decisions are often household decisions and that the core variable social capital is the household’s social capital rather than the individual’s social capital. The personal characteristics in the control variables refer to the personal characteristics of the head of the household (the respondent).

**TABLE 3:** Variable definitions, 2015 CHFS data.

**Variable**	**Definition**
Dependent variable	
Entrepreneurship	A dummy variable equal to one if any family member chose to be an entrepreneur and zero otherwise
Financial literacy score	The number of financial literacy questions that the respondents answered correctly
Financial literacy index	The financial literacy index that we construct
Variable of interest	
Social capital	The sum of expenditures on developing relationships (one thousand Yuan)
Community social capital	The average social capital of the community except the focal household
Control variables	
Individual characteristics	
Gender	A dummy variable equal to one if the household head is male and zero otherwise
Age	The age of the household head
Age^2^	Age squared
Education	The number of years of schooling of the household head
Married	A dummy variable equal to one if the household head is married and zero otherwise
Residence	A dummy variable equal to one if the household head holds local hukou and zero otherwise; hukou is the residence registration system related to individuals’ social status, and under the hukou system, rural residents have limited access to numerous types of public welfare goods and services
Servant	A dummy variable equal to one if the household head or the spouse is a servant and zero otherwise
Risk appetite	The risk preference of the household head equal to five if the household head prefers projects with low risk and low returns and equal to one if the household head prefers projects with high risk and high returns; based on the question “Which of the options below do you want to invest in the most if you have adequate money?”
Household characteristics	
Asset	Household net assets per capita last year (one thousand Yuan)
Income	Household net income per capita last year (one thousand Yuan)
Family Size	The number of family members
Seniors	The number of seniors (>60 years old) in the family
Kids	The number of kids (<12 years old) in the family
Provincial characteristics	
ICTs adoption	Proportion of Internet users to total population in the province

We test the assumptions that the model depends on. First, the sample is randomly selected. The sampling is performed according to the principle of uniform sample selection and using the probability proportional to size (PPS) sampling method. Second, we check for normal distribution. These statistics are found to be in the acceptable range. Third, we use robust standard errors in our estimation to control the heteroscedasticity. Fourth, we calculate the correlation coefficients to assure that there is no serious multicollinearity. Overall, the results based on the data and models used in the study are robust.

Based on the regression results, we can calculate the values, standard deviations and 95% confidence intervals of the mediating effects as follows:

(4) $$M\,e\,d\,i\,a\,t\,i\,n\,g\,E\,f\,f\,e\,c\,t=\beta_{1}\,\gamma_{2}$$

(5) $$S_{M\,E}=\sqrt{\beta_{1}^{2}\,S_{\gamma_{2}}^{2}+\gamma_{2}^{2}\,S_{\beta_{1}}^{2}}$$

(6) $$95\%\,C\,I=\beta_{1}\,\gamma_{2}\pm 1.96\,S_{M\,E}$$

When we divide the sample into different groups, we can compare the mediating effects.

The model setting may face endogeneity issues from two sources. The first is omitted variables. Families with more social capital may have some inherent qualities, such as the characteristics of successful people. In addition to family characteristics, regional culture cannot be ignored. However, these variables are very hard to quantify. We consider the problem of omitted variables when we select the independent variables. Thus, we add more control variables (Xiao and Wu, 2020). The cost is that there are some invalid regression variables that will make the variance of the estimated coefficients larger. The second source of endogeneity is reverse causality, which implies that entrepreneurship affects family social relationships in turn. To address this endogeneity issue, we use the average social capital of households in the same community (586 communities in rural areas) except the focal household as an instrumental variable. The community is the most basic unit of residence in China. A community is a neighborhood with clearly distinguishable psychological, economic, and geographical boundaries. The average social capital of the community can be used as an instrumental variable of households’ social capital for three reasons. First, a community has clear geographical boundaries. The lives of family members are linked to various social structures and are defined by the community in time and space. The social activities between families in a certain space are interactive (Lin, 2002). Second, the community is currently the most densely populated carrier of various grassroots organizations (e.g., homeowners’ committees, residents’ recreational organizations). Frequent interactions and close relationships among households are usually formed in the community. Third, households have a strong psychological identification with the regional unit, forming a collective identity and even prompting collective actions. We then use two-stage least squares (2SLS) regression to alleviate endogeneity.

### Descriptive Statistics

As shown in Table 4, the proportion of entrepreneurship among rural households is 10.8%. The average financial literacy score is 0.545, and the average financial literacy index is −0.491. We explain the economic meaning of the financial literacy index as follows. Respondents do not know how to calculate interest rate. Only 35% of respondents know about interest rate and only 16% are correct. Respondents also fail to understand inflation. Only 39% of respondents know about inflation and the proportion of correct answers is lower (13%). Similarly, respondents display difficulty in grasping the concept of risk awareness. Less than 30% of them heard about stock and funds and only 27% know the difference between stock and funds. The mean value of social capital indicates that the household spends an average of 27,590 CNY on developing relationships. The sample shows that 61.9% of household heads are male, and most of them have a primary educational level. In rural areas, 95.5% of household heads are married, and they are extremely risk averse. The average net asset is 205,920 CNY, and the average net income is 26,031 CNY.

**TABLE 4:** Descriptive statistics of the variables.

**Variable**	**Obs**	**Mean**	**S.D.**	**Max**	**Min**
Entrepreneurship	11,654	0.108	0.31	1	0
Financial literacy score	11,654	0.545	0.757	3	0
Financial literacy index	11,654	−0.491	0.855	1.326	−1.282
Social capital	11,654	2.759	4.303	120.85	0
Community social capital	11,654	2.779	1.787	15.068	0.106
Gender	11,654	0.619	0.486	1	0
Age	11,654	54.128	13.59	83	19
Age^2^	11,654	3,114.561	1,451.706	6,889	361
Education	11,654	6.709	3.701	16	0
Married	11,654	0.955	0.207	1	0
Residence	11,654	0.987	0.111	1	0
Servant	11,654	0.007	0.084	1	0
Risk appetite	11,654	4.313	1.057	5	1
Asset	11,654	205.92	335.833	2,166.417	−49.29
Income	11,654	26.031	38.517	212.953	−7.077
Family size	11,654	1.819	1.521	7	1
Seniors	11,654	0.834	0.864	3	0
Kids	11,654	0.498	0.774	3	0
ICTs adoption	29	62.874	10.193	85.21	49.24

## Results and Discussion

We provide the correlation coefficient matrix of the variables in Table 5 as a preliminary analysis. The results show that there is a significant positive correlation between the core variables social capital, financial literacy and entrepreneurship, which implies that further regression analysis is necessary. The correlation coefficient between the financial literacy score and the financial literacy index is 0.81 and significant at the 1% level. This indicates that each one can be used as a robustness test. Social capital and community social capital are significantly and positively correlated, indicating that the instrumental variable is valid.

**TABLE 5:** Correlation coefficient matrix of the variables.

	**Entrepreneurship**	**Financial**	**Financial**	**Capital**	**Community**	**Gender**	**Age**	**Age^2^**	**Education**	**Married**	**Residence**	**Servant**	**Risk**	**Asset**	**Income**	**Family**	**Seniors**	**Kids**	**ICTs**
	****	**literacy**	**literacy**	**Social**	**Capital**	****	****	****	****	****	****	****	**Appetite**	****	****	**Size**	****	****	**adoption**
	****	**score**	**index**	**Capital**	****	****	****	****	****	****	****	****	****	****	****	****	****	****	****
Entrepreneurship	1.00																		
Financial literacy score	0.09*	1.00																	
Financial literacy index	0.12*	0.81*	1.00																
Social capital	0.15*	0.18*	0.21*	1.00															
Community social capital	0.07*	0.24*	0.30*	0.28*	1.000														
Gender	0.01*	0.02*	0.01*	0.00	−0.06*	1.00													
Age	−0.20*	−0.29*	−0.31*	−0.13*	−0.10*	0.06*	1.00												
Age^2^	−0.20*	−0.27*	−0.30*	−0.13*	−0.09*	0.06*	0.98*	1.00											
Education	0.08*	0.45*	0.53*	0.23*	0.31*	0.07*	−0.39*	−0.37*	1.00										
Married	−0.02*	−0.11*	−0.12*	0.00	−0.06*	−0.04*	0.31*	0.26*	−0.17*	1.00									
Residence	−0.11*	−0.12*	−0.14*	−0.09*	−0.20*	0.01*	0.23*	0.21*	−0.15*	0.13*	1.00								
Servant	−0.01*	0.08*	0.09*	0.07*	0.08*	−0.02*	−0.05*	−0.05*	0.15*	0.00	0.00	1.00							
Risk appetite	−0.12*	−0.29*	−0.30*	−0.15*	−0.12*	−0.08*	0.34*	0.32*	−0.28*	0.16*	0.11*	−0.06*	1.00						
Asset	0.15*	0.23*	0.27*	0.28*	0.28*	–0.00	−0.04*	−0.03*	0.28*	−0.01*	−0.03*	0.07*	−0.15*	1.00					
Income	0.15*	0.25*	0.28*	0.29*	0.21*	0.01*	−0.13*	−0.13*	0.31*	−0.01*	−0.07*	0.10*	−0.19*	0.56*	1.00				
Family size	0.07*	0.01*	0.01	0.06*	0.08*	0.02*	−0.10*	−0.11*	0.02*	0.05*	−0.06*	0.00	−0.03*	−0.19*	−0.23*	1.00			
Seniors	−0.12*	−0.14*	−0.16*	−0.10*	−0.10*	0.08*	0.56*	0.59*	−0.22*	0.12*	0.14*	−0.06*	0.18*	–0.00	−0.06*	−0.03*	1.00		
Kids	0.10*	–0.00	−0.02*	0.01*	−0.06*	–0.00	−0.21*	−0.21*	−0.03*	0.13*	−0.02*	−0.01*	−0.06*	−0.03*	0.01*	0.19*	−0.07*	1.00	
ICTs adoption	0.01*	−0.05*	−0.07*	–0.00	−0.02*	0.01	0.02*	0.01*	−0.07*	0.00	0.01	0.01*	0.00	−0.03*	–0.00	−0.07*	0.01*	0.05*	1.00

*^∗^*p* < 0.01.*

Table 6 shows the results of comparing the variables between groups. Entrepreneurs have significantly higher levels of financial literacy than non-entrepreneurs (0.78 vs. 0.51), which indicates the important role of financial literacy in entrepreneurship. The mean value of social capital differs significantly between entrepreneurs and non-entrepreneurs. Entrepreneurs have significantly more social capital than non-entrepreneurs (4.895 vs. 2.501). After merging ICTs adoption data with household data, we find that provinces with higher ICTs adoption rates are more conducive to entrepreneurship (63.371 vs. 62.814).

**TABLE 6:** Comparison of the variables between different groups.

**Variable**	**Entrepreneurship = 1**		**Entrepreneurship = 0**		
	**Mean**	**S.D.**	**Mean**	**S.D.**	**T-statistics**
Financial literacy score	0.78	0.837	0.517	0.742	11.7***
Financial literacy index	−0.158	0.897	−0.531	0.841	14.75***
Social Capital	4.895	6.906	2.501	3.792	18.9***
Community social capital	3.162	2.001	2.733	1.754	8.05***
Gender	0.647	0.478	0.615	0.487	2.2**
Age	49.25	12.55	54.717	13.593	−13.55***
Age^2^	2,582.99	1,262.92	3,178.65	1,459.86	−13.85***
Education	7.962	3.342	6.558	3.713	12.8***
Married	0.959	0.199	0.955	0.208	0.6
Residence	0.978	0.145	0.989	0.106	−3.05***
Servant	0.01	0.101	0.007	0.081	1.5
Risk Appetite	4.061	1.194	4.343	1.035	−8.95***
Asset	500.726	595.625	170.373	268.129	34.55***
Income	48.304	57.683	23.346	34.562	22.15***
Family Size	2.103	1.809	1.785	1.479	7***
Seniors	0.668	0.842	0.853	0.864	−7.2***
Kids	0.629	0.814	0.482	0.768	6.4***
ICTs adoption	63.371	9.794	62.814	10.239	1.85*
Observations	1,254		10,400		

***p < 0.01; **p < 0.05; and *p < 0.1.

Regarding other control variables, entrepreneurs are more likely to be male and younger than non-entrepreneurs. In addition, entrepreneurs are more educated (7.962 vs. 6.588 years), and they prefer to take more risk (4.061 vs. 4.343), suggesting that entrepreneurship is risky and requires knowledge. Entrepreneurs are significantly wealthier than non-entrepreneurs.

Table 7 shows the estimation results of the relationships between social capital, financial literacy and entrepreneurship. The first part of the table (column 1) refers to the results of the effect of social capital on entrepreneurship. The second part of the table (columns 2–3) refers to the results of the effect of social capital on financial literacy. Finally, the third part of the table (columns 4–5) refers to the results of the partial mediating effect of financial literacy. The regression results show that the effect of social capital on entrepreneurship is significantly positive. Similarly, social capital significantly affects financial literacy. Furthermore, after adding financial literacy to the regression, we find a partial mediating effect: social capital promotes household entrepreneurship through sharing financial literacy. Therefore, Hypothesis 1 holds.

**TABLE 7:** Estimation of the effect of social capital on entrepreneurship and financial literacy.

**Dependent Variable**	**(1) Entrepreneurship**	**(2) Financial literacy score**	**(3) Financial literacy index**	**(4) Entrepreneurship**	**(5) Entrepreneurship**
Social capital	0.023***	0.008***	0.012***	0.022***	0.022***
	(0.004)	(0.002)	(0.002)	(0.004)	(0.004)
Financial literacy score				0.060***	
				(0.023)	
Financial literacy index					0.088***
					(0.022)
Gender	−0.013	0.056***	0.078***	−0.016	−0.019
	(0.037)	(0.014)	(0.015)	(0.037)	(0.037)
Age	0.008	−0.015***	−0.015***	0.009	0.009
	(0.008)	(0.003)	(0.003)	(0.008)	(0.008)
Age^2^	−0.000**	0.000**	0.000	−0.000**	−0.000**
	(0.000)	(0.000)	(0.000)	(0.000)	(0.000)
Education	0.021***	0.038***	0.051***	0.018***	0.015***
	(0.005)	(0.002)	(0.002)	(0.005)	(0.005)
Married	0.111	0.005	−0.001	0.110	0.110
	(0.092)	(0.034)	(0.037)	(0.092)	(0.092)
Residence	−0.192	0.035	0.040	−0.191	−0.194
	(0.128)	(0.060)	(0.061)	(0.128)	(0.128)
Servant	−0.173	0.025	0.181*	−0.173	−0.186
	(0.206)	(0.081)	(0.095)	(0.206)	(0.207)
Risk appetite	−0.029*	−0.099***	−0.124***	−0.023	−0.017
	(0.016)	(0.007)	(0.007)	(0.016)	(0.016)
Asset	0.001***	0.000***	0.000***	0.001***	0.001***
	(0.000)	(0.000)	(0.000)	(0.000)	(0.000)
Income	0.003***	0.001***	0.001***	0.003***	0.003***
	(0.000)	(0.000)	(0.000)	(0.000)	(0.000)
Family size	0.126***	0.018***	0.015***	0.125***	0.125***
	(0.011)	(0.005)	(0.005)	(0.011)	(0.011)
Seniors	0.018	0.007	0.021**	0.017	0.015
	(0.024)	(0.009)	(0.010)	(0.024)	(0.024)
Kids	0.020	−0.006	−0.022**	0.020	0.022
	(0.023)	(0.009)	(0.009)	(0.023)	(0.023)
Province fixed effect	YES	YES	YES	YES	YES
Observations	11,654	11,654	11,654	11,654	11,654
Pseudo/R-squared	0.160	0.167	0.241	0.161	0.163

Estimated marginal effects are reported. Standard errors are in parentheses. ***p < 0.01; **p < 0.05; and *p < 0.1.

We interpret the results as follows. Due to the scarcity of human capital and the immature market mechanism in rural areas, households pay more attention to the role of social relationships. Social networks among friends play a significant role in entrepreneurship, serving as an informal information source. By increasing expenditures on developing relationships, rural households interact more frequently with individuals in their social networks. They learn knowledge about entrepreneurship, such as financial knowledge, through increased social interactions. Thus, social capital becomes an important way to share the financial knowledge needed to start a business.

Regarding the control variables, households with a higher level of education are more likely to become entrepreneurs. Entrepreneurship is a creative activity and entails various difficulties. A certain level of knowledge helps entrepreneurs find business opportunities and overcome challenges (Lussier and Pfeifer, 2001). At the same time, in the process of entrepreneurship, continuous learning is a necessary quality for successful entrepreneurs (Vakili et al., 2016). In addition, risk appetite has a direct impact on entrepreneurial behavior, and families who are risk takers are more likely to accept the uncertainty of self-employment. They are adept at using various financial instruments to mitigate financial problems (Fang and An, 2017). Financial literacy can also change households’ risk appetite; thus, the coefficient of risk appetite in columns 4–5 is not significant. Household net assets and income can increase the probability of starting a business, which is consistent with other studies. A lack of capital is a common obstacle faced by entrepreneurs worldwide (Lelarge et al., 2010). Due to adverse selection and moral hazard, the capital market does not provide enough capital to entrepreneurs, who still need their household’s accumulated wealth to realize entrepreneurship (Moskowitz and Vissing-Jørgensen, 2002; Fairlie and Krashinsky, 2012).

Entrepreneurship affects household consumption and saving behavior (Cai et al., 2018). Entrepreneurs reduce their saving rate while increasing their social spending, which in some ways expands their own social network. Therefore, it is necessary to find a suitable instrumental variable for social capital. We choose average community social capital as an instrumental variable for household social capital. Average community social capital is correlated with household social capital but not with household entrepreneurship. Table 8 presents the instrumental variable regression results of the effect of social capital on entrepreneurship and financial literacy. In the first-stage regression, community social capital is significantly and positively related to household social capital. In the second-stage regression, social capital significantly promotes entrepreneurship and shares financial literacy, which is consistent with the previous results.

**TABLE 8:** IV estimation of the effect of social capital on entrepreneurship and financial literacy.

**Dependent variable**	**(1) Entrepreneurship**	**(2) Financial literacy score**	**(3) Financial literacy index**	**(4) Entrepreneurship**	**(5) Entrepreneurship**	**(6) First-stage social capital**
Social capital	0.054**	0.033***	0.059***	0.053**	0.050**	
	(0.025)	(0.011)	(0.012)	(0.025)	(0.025)	
Financial literacy score				0.052**		
				(0.024)		
Financial literacy index					0.078***	
					(0.024)	
Gender	−0.008	0.059***	0.084***	−0.012	−0.015	−0.075
	(0.037)	(0.014)	(0.016)	(0.037)	(0.037)	(0.079)
Age	0.006	−0.017***	−0.018***	0.007	0.007	0.070***
	(0.009)	(0.003)	(0.004)	(0.009)	(0.009)	(0.014)
Age^2^	−0.000*	0.000***	0.000**	−0.000*	−0.000*	−0.001***
	(0.000)	(0.000)	(0.000)	(0.000)	(0.000)	(0.000)
Education	0.018***	0.036***	0.047***	0.016***	0.014**	0.079***
	(0.006)	(0.002)	(0.002)	(0.006)	(0.006)	(0.011)
Married	0.085	−0.014	−0.038	0.084	0.087	0.780***
	(0.093)	(0.035)	(0.038)	(0.093)	(0.094)	(0.137)
Residence	−0.192	0.034	0.037	−0.192	−0.194	0.070
	(0.127)	(0.060)	(0.063)	(0.127)	(0.128)	(0.306)
Servant	−0.215	−0.007	0.120	−0.214	−0.222	1.238
	(0.215)	(0.085)	(0.108)	(0.215)	(0.215)	(0.949)
Risk appetite	−0.023	−0.095***	−0.116***	−0.018	−0.013	−0.170***
	(0.016)	(0.007)	(0.008)	(0.016)	(0.016)	(0.041)
Asset	0.001***	0.000***	0.000***	0.001***	0.001***	0.002***
	(0.000)	(0.000)	(0.000)	(0.000)	(0.000)	(0.000)
Income	0.003***	0.000	0.000*	0.003***	0.003***	0.013***
	(0.001)	(0.000)	(0.000)	(0.001)	(0.001)	(0.002)
Family size	0.113***	0.009	−0.003	0.113***	0.114***	0.329***
	(0.016)	(0.006)	(0.007)	(0.016)	(0.016)	(0.028)
Seniors	0.027	0.014	0.034***	0.026	0.024	−0.220***
	(0.024)	(0.009)	(0.010)	(0.024)	(0.024)	(0.046)
Kids	0.025	−0.002	−0.015	0.025	0.026	−0.111**
	(0.023)	(0.009)	(0.010)	(0.023)	(0.023)	(0.048)
Community social capital						0.441***
						(0.035)
Province fixed effect	YES	YES	YES	YES	YES	YES
Observations	11,654	11,654	11,654	11,654	11,654	11,654
Pseudo/R-squared	0.156	0.166	0.240	0.107	0.109	0.180

*Estimated marginal effects are reported. Standard errors are in parentheses. ^∗∗∗^*p* < 0.01, ^∗∗^*p* < 0.05, and ^∗^*p* < 0.1.*

Next, we divide the sample into three groups according to the ranking of the provincial level of ICTs adoption. The grouping criterion was a cutoff of 55 and 64% to have as many samples as possible in each group. For each group, we calculate the value of the estimated mediating effect and provide 95% confidence intervals (see Table 9). We find that the mediating effect increases as ICTs adoption increases. In the provinces with the lowest level of ICTs adoption (below 55%), the mediating effect is not even significant at the 5% level. The application of ICTs expands the boundaries of social networks, and rural households can use social capital more efficiently to obtain the financial literacy needed for entrepreneurship. Therefore, Hypothesis 2 holds.

**TABLE 9:** Mediating effects at different levels of ICTs adoption.

**Variable**	**Financial literacy score**			**Financial literacy index**		
	**Value**	**S.D.**	**95% CI**	**Value**	**S.D.**	**95% CI**
Full sample (Obs = 11,645)						
Basic estimation	0.0004	0.0002	(0.0000, 0.0008)	0.0010	0.0003	(0.0004, 0.0016)
IV estimation	0.0017	0.0010	(−0.0002, 0.0037)	0.0029	0.0016	(−0.0001, 0.0061)
Low (Obs = 3,821)						
Basic estimation	0.0000	0.0001	(−0.0002, 0.0003)	0.0003	0.0004	(−0.0005, 0.0013)
IV estimation	0.0005	0.0005	(−0.0004, 0.0014)	0.0009	0.0010	(−0.0010, 0.0028)
Medium (Obs = 3,613)						
Basic estimation	0.0006	0.0003	(0.0000, 0.0012)	0.0012	0.0006	(0.0000, 0.0024)
IV estimation	0.0010	0.0006	(−0.0001, 0.0021)	0.0023	0.0012	(−0.0000, 0.0046)
High (Obs = 4,220)						
Basic estimation	0.0007	0.0003	(0.0000, 0.0013)	0.0013	0.0005	(0.0003, 0.0023)
IV estimation	0.0025	0.0024	(0.0005, 0.0044)	0.0057	0.0025	(0.0008, 0.0106)

## Conclusion

Encouraging rural entrepreneurship lifts rural households out of poverty. A certain level of financial literacy is a necessary skill for entrepreneurs. For rural households, social capital is an important resource for achieving entrepreneurship. Using 2015 CHFS data, we analyze the effect of social capital on rural entrepreneurship and the mediating effect of financial literacy with the help of ICTs.

In this study, we select household expenditures on developing relationships to construct a social capital index, as this is a widespread form of bridging social capital. We then construct two indicators to measure financial literacy based on households’ responses to financial knowledge questions. The empirical results show in rural areas that households with more social capital tend to become entrepreneurs. Social capital promotes rural entrepreneurship through different mechanisms. One of them is that social capital shares financial literacy. It also shows that rural households learn knowledge from their social networks. Furthermore, this mediating effect is stronger in regions with a high level of ICTs adoption.

Rural entrepreneurial conditions are poorly supported by local resources. Tangible assets are relatively easy to acquire, but intangible assets, such as knowledge, are not easily transferred to proper places. Rural households rely on their social networks to obtain knowledge. Engaging in social interactions and communicating with others in their social networks help rural households improve their financial literacy and thus start a business. Therefore, the role of social capital in promoting rural entrepreneurship cannot be ignored. ICTs enable rural households to interact online, broadening social networks, which allows them to learn more efficiently through social capital. The results imply the positive effect of ICTs adoption on rural entrepreneurship.

## Recommendations

The results suggest the following recommendations. First, the government should pay attention to the role of social capital in knowledge sharing and encourage individuals with special knowledge to share it. The empirical analysis shows that rural households acquire financial knowledge through social interaction to achieve entrepreneurship. The government can establish an incentive mechanism to reward individuals or organizations that contribute to financial and entrepreneurial knowledge sharing. The knowledge sharing incentive mechanism facilitates knowledge sharing in social networks, thus promoting rural entrepreneurship. Second, establishing specific interactive contexts can improve the efficiency of knowledge sharing. The government can hold regular or irregular lectures on financial literacy and provide timely training on entrepreneurship, which prepares rural households for knowledge sharing and improves their literacy level. Third, the government can play a role by ensuring that ICTs infrastructure constraints do not limit the progress of rural entrepreneurship. Due to the low quantity and low quality of rural infrastructure supply, entrepreneurial activity in rural areas could be limited by communication infrastructure. To achieve high knowledge acquisition efficiency, potential entrepreneurs in rural areas must have access to ICTs. Through investment and policy, the government can build communication infrastructure in rural areas to create an environment where entrepreneurship can thrive.

## Limitations

There are some limitations in this study. First, this study does not use a cross-country sample for empirical analysis. Social capital has the attributes of culture and tradition, and the way of developing social relationships differs among countries. Therefore, whether the social capital index in this paper is applicable to other countries remains to be studied. Further research using cross-country data for the analysis modifies the bias caused by culture and tradition. Second, we group the sample according to provincial ICTs adoption level in the heterogeneous analysis of ICTs adoption, which does not represent the specific situation of ICTs usage among households. Unfortunately, we do not have data related to household use of ICTs. Our conclusions about the development of regional ICTs are still credible. Third, the issue of individual heterogeneity is not well addressed. The current empirical analysis is based on cross-sectional data, and the conclusions are reliable based on the assumption that the sample is homogeneous. However, the assumption of a homogeneous sample is not realistic. Although we add many levels of control variables to measure household heterogeneity in the empirical analysis, this only alleviates the problem of individual heterogeneity. Panel data are needed because panel data capture the dynamics of rural entrepreneurship (i.e., the process by which non-entrepreneurial households turn into entrepreneurial households). By using panel data, the results would become more convincing.

## Data Availability Statement

The original contributions presented in the study are included in the article/Supplementary Material, further inquiries can be directed to the corresponding author/s.

## Ethics Statement

Ethical review and approval was not required for the study on human participants in accordance with the local legislation and institutional requirements. Written informed consent for participation was not required for this study in accordance with the national legislation and the institutional requirements.

## Author Contributions

JZ and TL designed and performed the research and wrote the manuscript. TL analyzed the data. Both authors contributed to the article and approved the submitted version.

## Conflict of Interest

The authors declare that the research was conducted in the absence of any commercial or financial relationships that could be construed as a potential conflict of interest.

## Publisher’s Note

All claims expressed in this article are solely those of the authors and do not necessarily represent those of their affiliated organizations, or those of the publisher, the editors and the reviewers. Any product that may be evaluated in this article, or claim that may be made by its manufacturer, is not guaranteed or endorsed by the publisher.
